# PP39 Monitoring Clinical Trial Registers In The Context Of A Systematic Review On Vitamin D For COVID-19

**DOI:** 10.1017/S0266462325102584

**Published:** 2025-12-29

**Authors:** Trinidad Sabalete-Moya, Rebeca Isabel-Gómez, Juan Antonio Blasco Amaro

## Abstract

**Introduction:**

Clinical trial registries provide relevant information to address publication bias in systematic reviews. However, monitoring these registries is a complex process. Results are rarely posted in registries, and finding publications in journals requires a laborious search across multiple resources. We aimed to monitor registries of randomized clinical trials, results posted on platforms, and articles published in journals in the context of a rapid review to inform decision-makers.

**Methods:**

We searched ClinicalTrials.gov and the World Health Organization International Clinical Trials Registry Platform from April 2020 to November 2024 as part of a systematic search on the use of vitamin D in COVID-19, including the MEDLINE, Embase, Web of Science, and the Cochrane Library databases. We collected data such as the study start date, sample size, principal trial characteristics, population, intervention, control, and main outcome. We reviewed new register monitoring results published in databases or platforms and assessed each report’s update frequency until November 2024.

**Results:**

We found 54 unique register numbers with this different status information: 29 trials were completed, two terminated, four active (one recruiting and three not yet recruiting), two were ongoing, one had recruitment and regulatory approvals pending, 11 had unknown status, one was prematurely ended, and two were withdrawn. Two clinical trials were matched with one study published. In total, 26 trials had results published in journals. However, only two trials posted results on the platform. In the last review, 25 trials remained without results, and related publications were not found.

**Conclusions:**

Searching clinical trial registries as part of a systematic review strategy is a necessary source for addressing publication bias. Monitoring clinical trial registries provides relevant information on the development and length of trials, identifying gaps and failures to assist research. Lack of updated information on registries, and finding accurate and worthy information about trials, can result in a time consuming and sometimes unsuccessful process.

